# Intraspecific Diversity in the Cold Stress Response of Transposable Elements in the Diatom *Leptocylindrus aporus*

**DOI:** 10.3390/genes11010009

**Published:** 2019-12-20

**Authors:** Aikaterini Pargana, Francesco Musacchia, Remo Sanges, Monia Teresa Russo, Maria Immacolata Ferrante, Chris Bowler, Adriana Zingone

**Affiliations:** 1 Stazione Zoologica Anton Dohrn, Villa Comunale, 80121 Napoli, Italy; francescomusacchia@gmail.com (F.M.); remo.sanges@gmail.com (R.S.); monia.russo@szn.it (M.T.R.); mariella.ferrante@szn.it (M.I.F.); zingone@szn.it (A.Z.); 2 Institut de Biologie de l’Ecole Normale Supérieure (IBENS), CNRS, INSERM, PSL Université Paris, 75005 Paris, France; cbowler@biologie.ens.fr

**Keywords:** transposable elements, diatoms, *Leptocylindrus aporus*, RNA sequencing, gene expression, stress response, temperature, intraspecific diversity, in-culture evolution

## Abstract

Transposable elements (TEs), activated as a response to unfavorable conditions, have been proposed to contribute to the generation of genetic and phenotypic diversity in diatoms. Here we explore the transcriptome of three warm water strains of the diatom *Leptocylindrus aporus*, and the possible involvement of TEs in their response to changing temperature conditions. At low temperature (13 °C) several stress response proteins were overexpressed, confirming low temperature to be unfavorable for *L. aporus*, while TE-related transcripts of the LTR retrotransposon superfamily were the most enriched transcripts. Their expression levels, as well as most of the stress-related proteins, were found to vary significantly among strains, and even within the same strains analysed at different times. The lack of overexpression after many months of culturing suggests a possible role of physiological plasticity in response to growth under controlled laboratory conditions. While further investigation on the possible central role of TEs in the diatom stress response is warranted, the strain-specific responses and possible role of in-culture evolution draw attention to the interplay between the high intraspecific variability and the physiological plasticity of diatoms, which can both contribute to the adaptation of a species to a wide range of conditions in the marine environment.

## 1. Introduction

Diatoms are among the most common types of phytoplankton and one of the most successful clades of eukaryotic, single-celled photosynthetic organisms in the modern ocean [1]. They are widespread in the plankton and benthos of freshwater, coastal and oceanic habitats and even in temporarily wet terrestrial environments. The ability of diatoms to thrive under many different conditions in the natural environment could depend on:

(1) their phenotypic plasticity, reflected in their physiological diversity that allows them to acclimate and face short-term environmental heterogeneity [2,3],

(2) intraspecific genetic variations leading to distinct populations, each with a rather narrow physiological tolerance and response (i.e., adapted populations); numerous cases of cryptic or pseudo-cryptic diatom species have recently been uncovered [4,5,6], and even populations within species that show particular distributions as a result of adaptation to specific ecological niches [7], (3) both of the above, as plasticity can be adaptive with respect to a function and may be altered by natural selection and ultimately become or facilitate adaptation.

In addition to natural environmental variability, diatoms used in experimental research are also exposed to another source of variability; they are removed from their natural environment, isolated and transferred to laboratory conditions in which evolutionary processes do not cease to occur [8]. Many cases of novel genetic variation have been shown after years of maintenance in culture collection [9,10,11]. Considering the high adaptability of diatoms, it could be hard to interpret the intra-specific algal diversity observed in related studies as the result of (a) adaptation to the natural environment from which strains were isolated or (b) evolutionary changes while being in culture.

Temperature is a very significant environmental variable that affects the physiological response of diatoms and ultimately determines their phenological patterns and geographic ranges [12,13,14], as well as one of the parameters that is usually kept constant in culture conditions. An increase in temperature leads to an increase in enzyme activity in metabolic processes, including photosynthesis and respiration, so the cells are expected to grow faster. Even algae obtained from cold habitats have shown at least short-term temperature optima for photosynthesis several degrees higher than the temperature at which they were growing. Low temperatures lead to the exact opposite direction so that, in order to survive, cells respond with an increase in enzyme synthesis [15,16]. A recent study on the molecular mechanisms of temperature acclimation and adaptation in warm and cold adapted strains of the marine diatom *Chaetoceros* concluded that evolutionary change in baseline gene expression is a key mechanism used by diatoms to adapt to different growth temperatures [17]. However, there are temperature limits, which differ for each species and even for strains of the same species isolated from warm or cold waters [8,13,14]. Temperatures close to their environmental extremes might activate stress-related pathways engaged in restoring cellular homeostasis. Temperatures that are too high may lead to denaturation and degradation of certain proteins [18], reduced functionality of the photosynthetic machinery [19], decreased RUBISCO activity, stimulated respiration [20], disturbed functions of cell membranes [21], and, thus, to reduction of cell growth. On the other hand, cold responses have been less explored and have mainly focused on the reactions and adaptations of high latitude and polar diatoms [22,23,24,25].

The cell stress response, including heat stress, is characterized initially by an increase in stress-inducible proteins [26], followed by the activation of enhanced tolerance mechanisms involving extensive reprogramming of gene expression and further modifications of metabolism. In diatoms, specific proteins related to cold acclimation or heat stress have been identified, such as antifreeze proteins (AFPs) in polar diatoms of the genus *Fragilariopsis* [27], the heat stress-responsive protein HI-5a in the marine species *Chaetoceros compressus* [28], and the heat shock protein 20 [29] and 70/90 [30] in the marine species *Ditylum brightwellii*. In addition, diatoms seem to have a high number of heat shock transcription factors, albeit of unknown function [31].

Yet another possible mechanism to adapt quickly to extreme environments consists of genome re-arrangements through the action of transposable elements (TEs) [32], which are DNA sequences with diverse structures able to move within the genome. Genes encoding reverse transcriptase, which copies RNA into cDNA codified by class 1 TEs or retrotransposons, have been found to be highly abundant and active in marine plankton assemblages [33]. In diatoms the long terminal repeat (LTR) retrotransposons superfamily makes up 90% of TEs in the genome of *Phaeodactylum tricornutum* and 58% in *Thalassiosira pseudonana*, and diatom-specific TEs belong to the so called CoDi I, CoDi II and GyDi lineages [32]. Deletions, mutations and epigenetic ‘defense’ mechanisms of the cell inactivate or silence most of the TEs, which are otherwise highly mutagenic when targeting protein coding genes [34,35]. However, as the “epi-transposon” hypothesis suggests [36], changing environments, including temperature changes [37,38], can lead to stress-induced breakdown of the epigenetic suppression of TEs. Once reactivated, TEs can alter gene expression patterns or even restructure the genome by inserting into promoters and enhancers, causing exon shuffling, sequence expansion, gene duplication, novel gene formation and expansion [39,40,41,42,43,44,45]. Such TE-induced mutations increase genetically and epigenetically-based phenotypic variation and have been associated with adaptation to the environment in numerous studies on different organisms [46,47], while two active, diatom-specific retrotransposons, namely Blackbeard and Surcouf, have been proposed to act as environmental sensors in the response to stress in the diatom *Phaeodactylum tricornutum* [32,47]. Thus, TEs could substantially increase genomic diversity and be a crucial element in the acclimation and even adaptation of diatoms to the ever-changing aquatic environment but when in a constant environment such as the lab, their suppression would have no effects and could hence become permanent through genetic changes, leading to the loss of the diatom adaptability.

*Leptocylindrus aporus* belongs to an ancient centric diatom lineage, Leptocylindraceae, with a broad distribution, common from polar to sub-tropical coastal regions, and is often a conspicuous component of diatom blooms [48,49]. It grows equally efficiently at medium and high temperature but less at low temperature, where it might experience physiological stress [50]. In the Gulf of Naples, *L. aporus* has been found to bloom in summer but the species is present during other seasons as well [6,51]. Indications of a cold adapted population resulted from a worldwide high throughput sequencing (HTS) metabarcoding analysis on Leptocylindraceae, offering a possible explanation of the species occurrence through the year [51]. In the current study, (i) we used RNA sequencing of multiple strains of *L. aporus* to explore expression differences in response to temperature changes, focusing on TEs. (ii) Intraspecific genetic diversity was assessed with a single-nucleotide polymorphism (SNP) analysis. In addition, in order to detect possible genetic changes in laboratory conditions for the oldest strain, an additional SNP analysis was conducted comparing present data to the already available transcriptome in the Marine Microbial Eukaryote Transcriptome Sequencing Project (MMETSP) [52]. (iii) The effects of experimental conditions were explored with shorter acclimation times as well as with recently isolated *L. aporus* strains using qPCR.

Our overall goal was to better understand how diatoms acclimate and possibly adapt to different environmental conditions, which is a requisite to interpret their changes in distribution and phenological patterns.

## 2. Materials and Methods 

### 2.1. Leptocylindrus aporus Strains

The six *L. aporus* strains (clonal cultures of *L. aporus*, i.e., initiated from a single cell) used in the current study were isolated from surface waters at the LTER-MC site (40°48.5′ N, 14°15′ E) in the Gulf of Naples (GoN, Mediterranean Sea) on different dates from 2010 to 2016 (Table 1). The cultures were kept in K medium [53] plus silica at 20 °C, under fluorescent light of 100 μmol photons m^−2^ s^−1^ with a photoperiod of 12:12 (light: dark). The first three strains, used for RNA sequencing (RNA-Seq), were identical based on the ITS r-DNA marker and were selected in a way that they would cover diversity in terms of seasonality and possibly in physiological characteristics detected by growth experiments. Following the results of RNA-Seq, new isolations were performed in order to obtain strains with a lower time of culture maintenance to be used in qPCR experiments. The new strains were also molecularly identified as *L aporus*.

### 2.2. Acclimation for RNA-seq

The three strains selected for RNA-seq were acclimated to three temperatures, 13 °C, 19 °C and 26 °C, at a light intensity of 100 μmol photons m^−2^ s^−1^ and a photoperiod of L:D, 12:12 (Table 2). The temperature values were chosen based on the range recorded in the GoN during the year (13.0–27.5 °C, [54]). Starting from the maintenance conditions at 20 °C, cultures were maintained for one week at intermediate temperatures, namely 15 °C and 23 °C (Figure S1). Subsequently, they were moved to 13°C and 26 °C and grown in triplicates under the same light conditions, along with one triplicate grown at 19 °C. The following acclimation period was maintained for at least three growth cycles or more until the growth curves were stabilized. Due to the different behavior of each strain, the acclimation duration varied between strains, yet based on their growth rates they were all considered equally acclimated in the end.

Cell density was quantified under the light microscope and chlorophyll fluorescence was measured daily using a Turner 10-005 fluorometer. Growth rate was calculated based on plots of the logarithmic values (base 10) of cells density and/or fluorescence over the days. K10, the slope calculated from the linear trendline applied to the plot, was used in the following equations, where k equals doublings per day and Ke equals the exponential growth rate [55]:

k (div./day) = 3.322/K10 (1)

and

Ke (day-1) = 0.6931 k(2)

### 2.3. RNA Extraction and Sequencing

Starter cultures (100 mL) with a concentration of 2000 cells/mL in exponential phase were transferred to 1-liter volume flasks and grown until a final concentration of 50,000 cells/mL, ensuring they were still at the exponential phase. Cultures were harvested by filtration on 47 mm MF-Millipore mixed cellulose membrane filters (1.2 μm pore size), which were stored in TRIzol Reagent. RNA extraction was performed as described in Nanjappa et al. (2017) [56].

To remove DNA contamination Roche DNase I recombinant, RNase-free (10 u/μL) was used and RNA was then purified using the QIAGEN RNeasy Mini Kit, following the manufacturer’s protocol. RNA samples were run on a Bioanalyzer 2100 RNA Nano LabChip (Agilent, Palo Alto, CA, USA) for qualitative control and sent for deep sequencing on an Illumina HiSeq2000 instrument (Illumina: San Diego, CA, USA) (paired-end, 50 bases) to the European Molecular Biology Laboratory (EMBL) Genomics Core Facilities.

### 2.4. Quality Check, Assembly and Annotation

The quality of the reads was checked using FastQC v0.11.3 (Babraham Bioinformatics) and the trimming of adapters was performed by Trimmomatic 0.33 [57] (Parameters: ILLUMINACLIP:TruSeq3-PE-2.fa:2:30:10:1:true, SLIDINGWINDOW:3:22, MINLEN:25). Paired-end reads were assembled with Trinity 2.0.6 [58] (Parameters: --normalize_reads --inchworm_cpu 15 --bflyHeapSpaceInit 24G --bflyHeapSpaceMax 200G --bflyCalculateCPU --CPU 15 --jaccard_clip --min_kmer_cov 2). CD-HIT-EST [59] was used in order to reduce the redundancy of isoforms that belong to the same gene, clustering together all the transcripts having a high percentage of sequence similarity (CD-HIT threshold of 95% identity) (Parameters: -c 0.95 -n 8). The reads were aligned to the transcripts generated by the assembler with Bowtie v.1.1. [60] (Parameters: -p 20 --maxins 500 --chunkmbs 10240 --seedlen 20 -a --fr --tryhard) and SAMtools idxstats [61] were used to count the mapped reads. EdgeR [62] and an in-house R script (Annocript DEA) [63,64] were used in order to calculate CPMs (count per million) and extract transcripts with a CPM greater than 1 for at least 2 samples, respectively. Annocript 1.1.2 [64] was executed for the annotation of the transcripts using Swiss-Prot [65], UniRef90 [66], the Conserved Domains Database (CDD) [67], Rfam [68] and the SILVA [69] database.

The completeness of the assembly and annotation was quantified using the software BUSCO v.2 using the “protists” set of lineage-specific single copy genes [70]. The BUSCO results were compared to the corresponding ones produced from the *L. aporus* transcriptome available in the Marine Microbial Eukaryote Transcriptome Sequencing Project, which was based on the single strain B651 [56].

### 2.5. Differential Expression Analysis

To assess the distance among samples, non-metric multidimensional scaling (NMDS) analysis was performed based on the expression values (CPMs) of all transcripts. Subsequently, we used EdgeR to evaluate the expression of the transcripts obtained and statistically distinguish the expression of the genes among the experiments. The following comparisons were performed: 13 vs. 19 °C, 19 vs. 26 °C, 13 vs. 26 °C. Different strains were used as replicates. Transcript counts were obtained using samtools idx. Raw counts were transformed in count per million (CPM) with the R library “edgeR”, followed by a normalization step with the functions *calcNormFactors*, *estimateCommonDisp* and *estimateTagwiseDisp*. For each couple of sample CPMs, we executed a paired t-test to avoid the effect of the differences among strains using the R function *exactTest*. Transcripts were considered differentially expressed when the FDR (false discovery rate) ≤ 0.05 and the FC (fold change) > 2. A complementary analysis was performed between strain couples (1A1 vs. B651, 1A1 vs. 3A6, B651 vs. 3A6) using the temperature conditions as replicates. Significantly differentially expressed (DE) transcripts with no annotation were manually blasted in the National Center for Biotechnology Information (NCBI) and CDD databases. All significantly DE transcripts were also annotated for KEGG pathways using GhostKOALA [71]. The KEGG pathway annotation results were further processed in a similar way as in Liang et al. 2019 [17]. KEGG pathways and Brite categories were selected from the following categories: metabolism, genetic information processing, environmental information processing, and cellular processes. A heatmap based on the log transformed expression values was made for the significantly DE transcripts between strains. Pathways with less than five annotated KEGG orthology identifiers (KO IDs) were removed.

### 2.6. Enrichment Analysis and Clustering of DE Transcripts

The gene ontology (GO) terms that were enriched in the list of DE transcripts with respect to the overall transcriptome were identified with the R prop.test (parameters: alternative=prop.alt) function. The parameters used were 10 as the minimum number of transcripts associated to a GO class and a cut-off of adjusted p-value equal to 0.1 in order to consider a class significant.

With the aim of investigating the different functions of genes related to the expression dynamics, a cluster analysis based on unsupervised classification was applied. The software MeV [72] was used for the clustering of DE transcripts using the k-means algorithm with the following parameters: extraction of fifteen clusters (k = 15), 500 clusters re-ordering iteration, Pearson uncentered as distance measure, calculation of medians (k-medians) instead of means.

### 2.7. SNP Analysis

**Figure 1:**
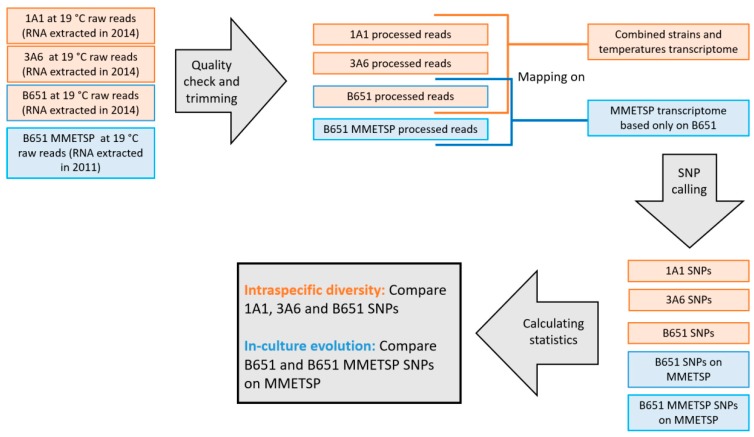
Workflow of the single nucleotide polymorphism (SNP) analysis on 1A1, 3A6 and B651 strains at 19 °C. An additional analysis (light blue boxes) was performed for B651, including the transcriptome publicly available in MMETSP B651, which is based on RNA extracted from the same strain but three years before the present experiment.

For the intraspecific diversity assessment, the quality checked and trimmed reads of all strains at 19 °C were mapped on the transcriptome using STAR 2.7.0c [73] and further processed with samtools (sorted and indexed). The software Opossum 0.2 was used in order to adjust the mapping of RNA-seq reads for variant calling [74]. The SNPs were called using FreeBayes v1.2.0-4-gd15209e [75] and filtered with bcftools by samtools, removing reads not properly mapped and with mapping quality less than 30. Variants with allele frequency (AF) equal or less than 0.2 were not considered (Figure 1).

To address possible variations over the culturing time, the same workflow was also followed mapping B651 reads acquired from this study and public B651 reads already available in MMETSP on the B651 MMETSP transcriptome. The B651 MMETSP reads and transcriptome are based on RNA extracted from the same B651 strain, at 19 °C in 2011, three years before the experiments described in this study. For this intra-strain comparison, the transcriptome used for the mapping was the B651 MMETSP transcriptome as it is based only on the B651 strain and would give more accurate results regarding the evolution through time of the strain itself, with no noise introduced by the other strains.

### 2.8. qPCR Validation of Gene Expression Levels from RNA-Seq

Following the investigation of the expression and function of all significantly DE genes, eight of them were selected as targets for validation and further experiments with qPCR (Genbank MN738467-MN738474). Five of the selected transcripts expressed TE-related genes and three expressed heat stress-related genes (Table 3). Tubulin A1 (TUBA1) and tubulin B6 (TUBB6) were tested for stable expression under the different experimental conditions using NormFinder software [76].

The samples used for validation with qPCR had undergone the same acclimation conditions and time as in the RNA-Seq experiment. The RNA was extracted following the same protocols described above and then reverse transcribed into cDNA using the QuantiTect Reverse Transcription Kit (Qiagen, Venlo, Limburg, the Netherlands). The specificity of all primers was checked by blasting against the whole *L. aporus* transcriptome. For the TE-related transcripts, a gradient PCR for a temperature range of 59.6–66.9 °C was conducted using randomly selected DNA from the studied strains. The reactions were performed in a final volume of 10 μl: cDNA 1μl, forward primer (10 μM), reverse primer (10 μM), PCR reaction buffer with MgCl2 (10×), dNTP (10×), Taq DNA Polymerase (5 u/μL). The products were run on 1.5% agarose gel in order to specify the size of the amplicon and confirm the presence of a single band, which demonstrates that a single region is amplified for each primer pair (Figure S2).

The efficiency of primers was calculated with an at least five point serial 10-fold dilution using the standard curve method of the ViiA™ 7 Real-Time PCR System (Applied Biosystems by Life Technologies, Carlsbad, CA, USA). The efficiency of all primers ranged between 1.85 and 2.41 (perfect efficiency equals 2, which means a double amount of DNA after one cycle). In addition, the melting curves, which ideally should show a single peak corresponding to a single product, were checked in order to verify the specificity of the primers already seen by the agarose gel. TR7186 showed a faint second band in the agarose gel but the qPCR melting curve did not detect any other product except for the targeted one. On the other hand, there were a few exceptions of melting curves that showed a smaller double peak indicating possible primer-dimers or non-specific amplification (TR6356, TR6586, TR6506_i5 in 1A1, 1188A1, 1189A3 and 1189B3 at 13 °C, HSFA in 1A1 at 26 °C). All these cases of extra peaks or slightly different peaks occurred when the amplification was low (see example Figure S3) and could have been related to the much lower level or absence of the target transcripts. This non-specificity would ultimately not have an important impact on the final interpretation of the low expression results. After verifying a satisfactory efficiency of the primers, qPCR was performed for all samples in triplicates with a negative control. Relative Expression Software Tool-Multiple Condition Solver (REST-MCS) was used for the calculation of the relative expression in qPCR. The software uses the pair-wise fixed reallocation randomization test [77]. The relative expression ratio of the targeted genes was computed as the expression variation between high temperature samples, set as control because *L. aporus* grows well at high temperature, against the low temperature samples, set as the stress condition, normalized over the expression variation of reference genes (TUBA1 and TUBB6) whose expression levels were not regulated in specific experimental conditions.

### 2.9. qPCR Experiments on Acclimation and Cultivation Time

To further explore the expression profile of the target genes in response to different acclimation and cultivation times, two qPCR experiments were designed and performed on two different sets of RNA samples, respectively (Table 4).

***Experiment 1***

In the first experiment, the same strains used in the RNA-Seq and validation were used but acclimation time was altered in order to investigate the acclimation duration impact. Strains were acclimated for a much shorter period compared with the time used in the RNA-Seq and validation experiments (39–66 days) and then used for qPCR analysis. These will be referred to as “_exp1” samples.

***Experiment 2***

To test the effect of time spent in culture, new *L. aporus* strains (1188A1, 1189A3, 1189B3) were isolated and used for qPCR after a short acclimation period (8–43 days). The acclimation duration varied due to the ease or difficulty of each strain that was acclimated (had a stable growth rate after three growth cycles).

In order to check the presence of the TE-related sequences in the genomes of all strains we also performed PCR on genomic DNA. Genomic DNA from strains used in both experiments was isolated using cetyltrimethylammonium bromide (CTAB). We added 500 μL CTAB and 12 μL β-mercaptoethanol to the cell pellet, then RNase was added and the reaction was incubated at 65 °C for 45 min. After addition of 500 μL SEVAG (chloroform: isoamyl alcohol 24:1), the sample was centrifuged at 20,000× *g* for 15 min at 4 °C. Precipitation occurred in 1 volume of ice-cold isopropanol at −20 °C and, after 2 washes in 75% ethanol, the pellet was air-dried and finally resuspended in water. PCRs were performed by using Wonder Taq DNA Polymerase (EuroClone) according to manufacturer’s instructions, with an annealing temperature of 57 °C and extension time of 1 min. Primer sequences were the same used for qPCR (Table 3).

### 2.10. Phylogenetic Analysis of the Selected TEs

The selected transcripts coding for transposable elements were screened for interspersed repeats and low complexity DNA sequences, like simple tandem repeats, polypurine and AT-rich regions, in the RepeatMasker (Search Engine: cross match, DNA source: diatom; [78]). The same screening was performed for the CoDi/GyDi sequences, which belong to groups of transposable elements mainly found in marine organisms with most of them being identified only in diatoms to date [32]. The CoDi/GyDi sequences were derived from Genbank using the Genbank accession numbers mentioned in [32]. The results of the two screenings were compared in order to identify whether the selected TEs represent known groups or new CoDis/GyDis. In addition, a phylogenetic analysis of the selected transcripts and the known CoDi/GyDi elements was performed. Multiple sequence alignment was performed with CLUSTAL Omega at EMBL-EBI [79]. Neighbor-joining trees were constructed for amino acid sequences with the bootstrap method set as the test of phylogeny (500 replications) and the Poisson model as a substitution model in MEGA7 [80] with GyDi group as outgroups, as was also done in [32]. All ambiguous positions were removed for each sequence pair. TR7186 and TR6586 were analysed separately in the phylogeny because it was not possible to estimate their pairwise distance.

## 3. Results

### 3.1. Leptocylindrus aporus Transcriptome

Preliminary growth experiments were performed on several *L. aporus* strains in order to identify the best candidates for investigating the species acclimation and adaptation using RNA-Seq (Supplementary File 1). Based on the V9 region included in the beginning of ITS marker, none of the strains belonged to the possible cold adapted population found in the worldwide HTS analysis [51]. Ultimately, one *L. aporus* strain isolated in summer 2010 (B651) and two isolated in winter 2013–2014 (1A1 and 3A6) were chosen, due to their diversity in terms of the year of isolation, seasonality and physiological characteristics. The final transcriptome of *L. aporus* (ArrayExpress ID: E-MTAB-8596), obtained using all results from the three strains grown at the three different temperatures, consisted of 19,963 transcripts. Further statistics are presented in Table S1.

The transcriptome obtained in the current study was compared to the previously available MMETSP transcriptome [52], obtained from the oldest strain B651, in order to test whether the set of 215 protist lineage-specific single-copy genes were present in the assemblies (BUSCO analysis). This analysis gave an indication on the completeness of the assemblies as well as their redundancy. While the two assemblies contained a similar number of complete genes from the dataset (100 for the current and 103 for the older assembly), the older assembly had a higher proportion of potentially erroneous assembled transcripts and therefore were classified as duplicated by BUSCO (7 for the current and 34 for the old) (Table S2).

### 3.2. Differential Expression: Heat and Cold Response

**Figure 2:**
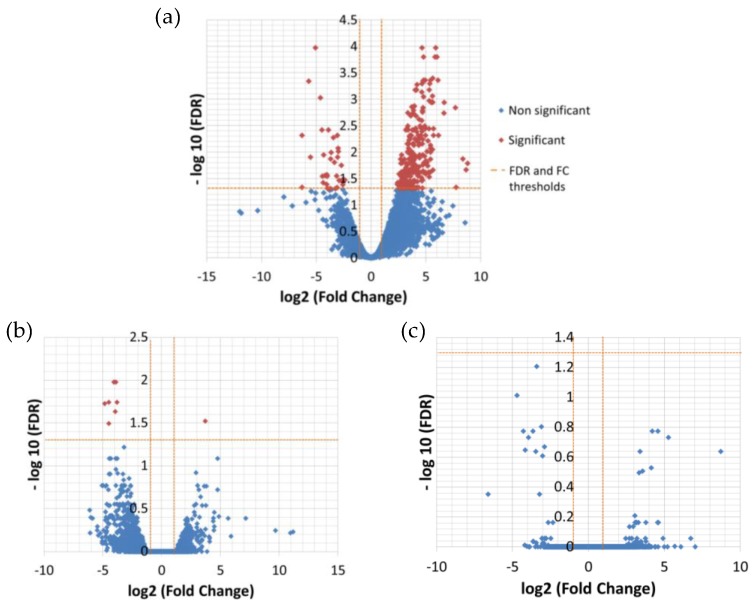
Volcano plots showing changes in gene expression between temperatures, cumulated for the three strains. The log10 of the statistical significance (FDR) is represented in the y-axis, while the x-axis shows the log fold-change between low and high temperature (**a**), medium and low temperature (**b**) and medium and high temperature (**c**). A FDR value of 0.05 and fold change of 2 are indicated by orange dashed lines. The significantly DE genes are shown in red.

The differential expression analysis between the temperatures resulted in 276 significantly DE transcripts between low (13 °C) and high (26 °C) temperature and only nine between low and medium (19 °C) temperature (Table 5, Figure 2), while there was no significant difference in expression between medium and high temperature (Supplementary File 2, 3 and 7).

All nine transcripts that were found to be significantly differentially expressed between low and medium temperature were also contained within the group of significantly differentially expressed genes in the low and high temperature contrast and followed the same direction of expression change as in the less extreme contrast.

A total of 49 significant DE transcripts, representing 17.7% of all DE transcripts, received an annotation related to temperature, referring to oxidative or any other kind of stress, heat/cold response and DNA integration, overall indicating a stress response of *L. aporus* at low temperature (Table 6).

**Figure 3:**
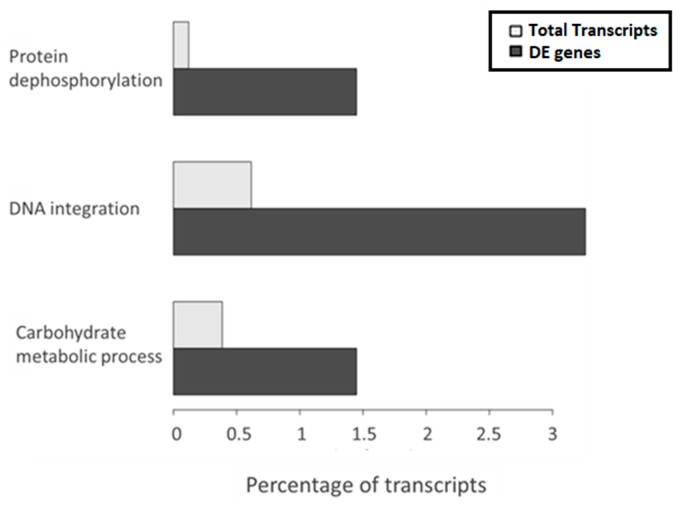
Biological process GO terms significantly enriched in the DE genes between high and low temperature.

The gene ontology (GO) enrichment analysis showed the GO terms enriched with respect to the overall transcriptome. The adjusted p-value cutoff to consider a class as significant was 0.1, which means that 10% of significant classes might be false positives. The analysis indicated that three classes belonging to the biological process GO aspect were significantly enriched among the DE transcripts between high and low temperature, protein dephosphorylation, DNA integration and carbohydrate metabolic process. Interestingly, among them, the most represented was DNA integration (Figure 3), which refers to the incorporation of a segment of DNA, including a transposon, into another, usually larger, DNA molecule such as a chromosome. Manual inspection of annotations revealed 16 DE transcripts related to DNA integration, four of which had no further annotation and 12 had specific protein annotation by at least one of the databases used in Annocript (Table 6). These were annotated as (1) reverse transcriptase (RNA-dependent DNA polymerase), which is usually indicative of a mobile element such as a retrotransposon or retrovirus, (2) ribonuclease of_Ty1/Copia or Ty3/Gypsy family, an endonuclease that cleaves the RNA strand of an RNA/DNA hybrid, which has been observed as adjunct domains to the reverse transcriptase gene in retroviruses, long-term repeat (LTR)-bearing and non-LTR retrotransposons, (3) integrase, which mediates integration of a DNA copy into the host chromosome, or (4) a gag-pol polyprotein, encoding structural proteins and several enzymatic functions in LTR retrotransposons.

All the significantly DE TE-related transcripts except one were highly expressed at low temperature in the recently isolated strains 1A1 and 3A6, while they were very low at all temperatures for the old B651 strain. This expression pattern was also the one mostly seen in several of the other stress-related transcripts (HSFs, SYM1, AOX4, ALDH, RaiA), and in three transcripts, manually annotated by blasting in NCBI and CDD databases as likely homologs of the bacterial MAI genes related to biomineralization, which are known to undergo frequent transposition events [81]. The observation of this expression pattern pointed towards a strain specific stress response. Indeed, in an NMDS analysis using the global gene expression matrix, the oldest strain B651 clustered apart from the other two strains, for which samples from the medium and high temperature were interspersed (Figure S4). All the above led to further investigation of the differences among strains.

**Figure 4:**
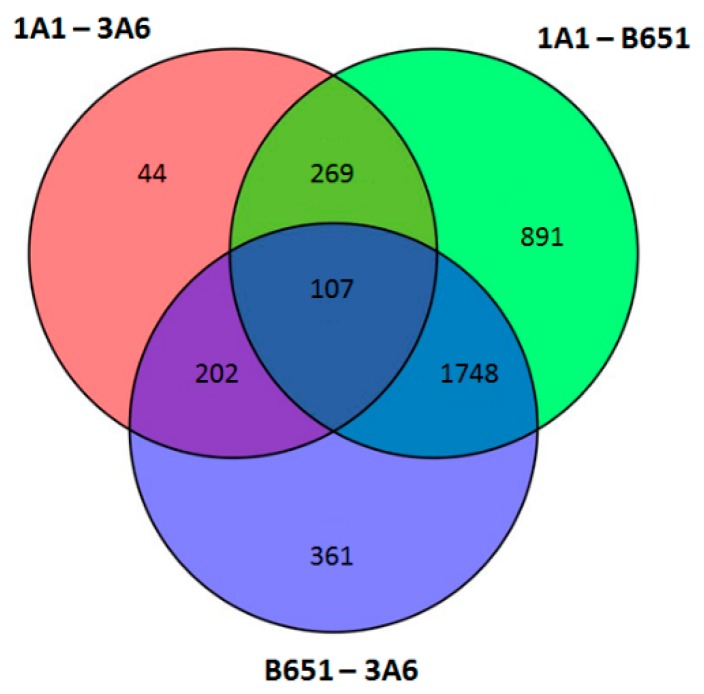
Venn diagram of the significantly differential expressed genes between strains in *L. aporus* when temperature conditions were used as replicates.

**Figure 5:**
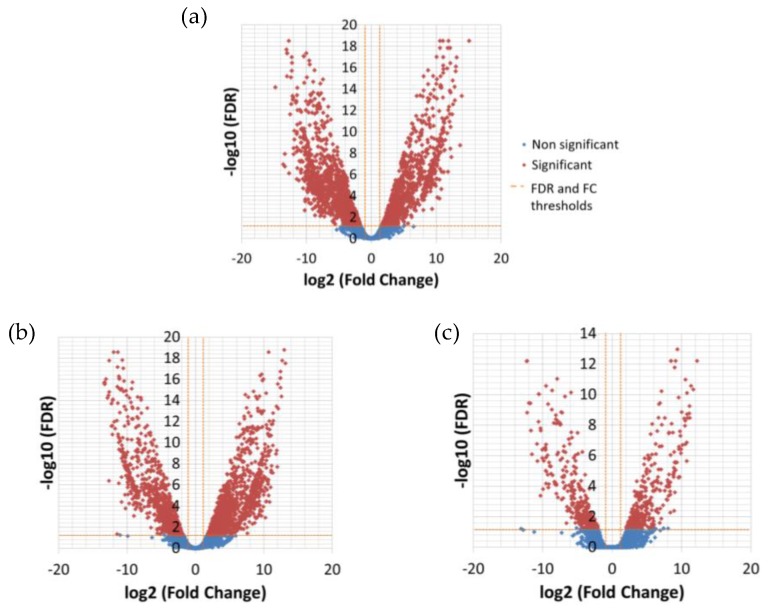
Volcano plots showing changes in gene expression between strains, cumulated for the three temperatures. The log10 of the statistical significance (FDR) is represented in the y-axis while the x-axis shows the log fold-change between the 1A1 and B651 strain (**a**), B651 and 3A6 strain (**b**) and 1A1 and 3A6 strain (**c**). A FDR value of 0.05 and fold change of 2 are indicated by orange dashed lines. The significantly DE genes are shown in red.

Considering couples of strains, significantly DE transcripts between 1A1 and 3A6 were 622; much less compared to the 3,015 between 1A1 and B651 and 2,418 between B651 and 3A6 (Figure 4 and Figure 5, Supplementary Files 4–7). 

DNA integration held a central role in this analysis as well, as it was the biological process that was significantly enriched in all three pairs of strains. The same TE-related transcripts detected in the temperature differential expression analysis were among the significantly DE ones from the strain analysis. Most of the DNA-integration transcripts were expressed in a similar manner, described above, i.e., higher at low temperature and lower in B651; there were even some transcripts completely absent from all B651 samples. Several key genes and pathways were included in the KEGG annotation of the significant DE transcripts among strains, with B651 clustering separately in the expression heatmap (Figure S5). Overall, the differential expression analysis among strains confirmed the B651-biased, TE-related expression pattern observed in the differential expression analysis among temperatures.

### 3.3. Clustering of DE Transcripts

**Figure 6:**
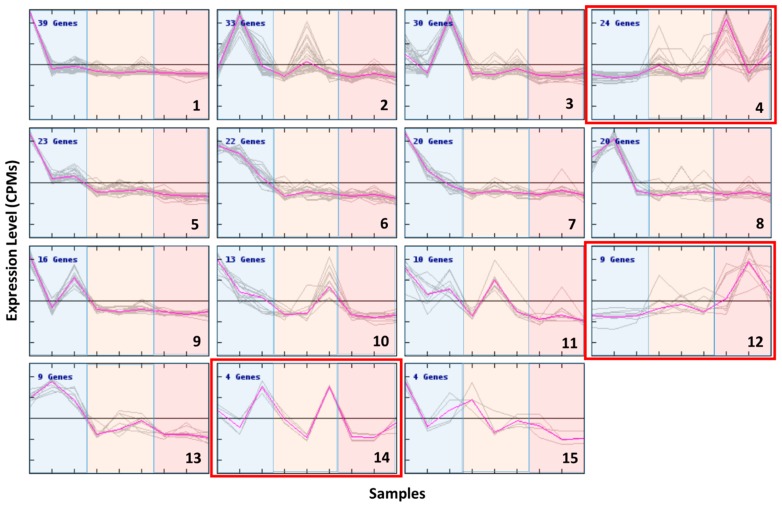
K-means clustering on the significant DE transcripts between temperatures. The dots in the bottom x-axis correspond to the samples (1A1-, B651-, 3A6-low temperature, 1A1-, B651-, 3A6- medium temperature, 1A1-, B651-, 3A6- high temperature). The low temperature samples are blue shadowed, the medium temperature ones are orange and the high temperature ones are red shadowed. Clusters are numbered on their bottom right corner while in each cluster the number of genes included is provided on the top left corner. Clusters 4, 12 and 14 (red borderline) are deviating from the main cold responsive trend.

Based on genes clustered by their expression patterns, the strongest cell response was to the cold conditions (Figure 6) except for three clusters (clusters 4, 12 and 14) showing an opposite pattern. At high temperature, cluster-4 genes were highly expressed only in the newer strains 3A6 and 1A1, whereas cluster-12 genes showed a higher level of expression in the old strain B651. Two transcripts related to TEs (transposase, ribonuclease) and a heat stress transcription factor (HSFC-1b) were present in cluster 4 and heat shock factor protein 1 in cluster 12.

Four out of the five TE-related genes of interest selected for qPCR (see below) were present in cluster 3, which, along with cluster 9, showed an increased expression at lower temperatures, but only for strains 3A6 and 1A1. At least seven more transcripts related to TEs or transposition (reverse transcriptase, ribonuclease, MAIs, gag-pol protein, integrase core domain)—eleven in total—and two heat stress-related transcripts (HSFA-1a, SYM1) were also present in these clusters. The GO terms that were significantly enriched in cluster 3 and 9 were DNA integration and antioxidant activity.

Cluster 2 included genes that showed an increase in expression while the temperature decreased but only in strain B651. The GO enrichment analysis in this cluster showed that oxidation–reduction process and DNA integration were significantly enriched terms. This cluster also included the remaining gene of interest that was investigated (TR6586) and two more TE-related transcripts.

Cluster 5 showed transcripts that were expressed more at cold temperature. This behavior was similar in all strains, but more pronounced in strain 1A1. DNA recombination and transport were the GO terms significantly enriched in this cluster.

Cluster 6 and 13 included transcripts highly expressed at low temperature for all strains. Heat stress transcription factor B-2a (HSFB-2a) was present in this cluster. The GO terms that were significantly enriched were metabolic process and catalytic activity.

Cluster 10 showed higher gene expression at low temperature for two strains B651 and 1A1, while in strain 3A6 the gene expression was lower at the extremes and higher at medium temperature. AOX was present in this cluster. The most significantly enriched GO term was oxidation–reduction process.

### 3.4. SNP Analysis and Genetic Diversity

The number of SNPs identified by FreeBayes was similar in 1A1 and 3A6, while it was almost double in the older strain B651 (Table 7). The heterozygous to homozygous ratio, for both total and SNP specific, was also quite different for B651 compared to 1A1 and 3A6.

The intra-strain SNP analysis of B651 showed that there were slightly fewer SNPs in the new transcriptome compared to the original MMETSP transcriptome. Furthermore, the total and SNP specific heterozygous to homozygous ratio has been reduced to half during the three years of culturing (Table 8).

### 3.5. Expression of Genes of Interest: Temperature and Strain Effects 

Five of the eight target genes used in qPCR were related to TEs (Table 9), as they were selected based on the most significantly enriched biological process GO term ‘DNA integration’.

The three remaining target genes were genes related to stress, as *L. aporus* seemed to go under stress at the low temperature.

### 3.6. qPCR Validation Results

The eight selected transcripts were cross-validated in the same strains, grown under the same conditions (validation set), and all of the results were found to be consistent with the results from the RNA-Seq samples with the single exception of HSFB in strain 1A1, which showed almost no change in expression (Figures S6 and S7).

As in RNA-Seq, B651 stood out based on its different regulation compared to 1A1 and 3A6. This was especially true in the case of HSFA, TR6506_i3 and TR6586: the former two were highly induced in 1A1 and 3A6 at low temperature but down or not regulated in B651, while the latter followed the opposite trend.

### 3.7. qPCR Results on Acclimation and Cultivation Time

**Figure 7:**
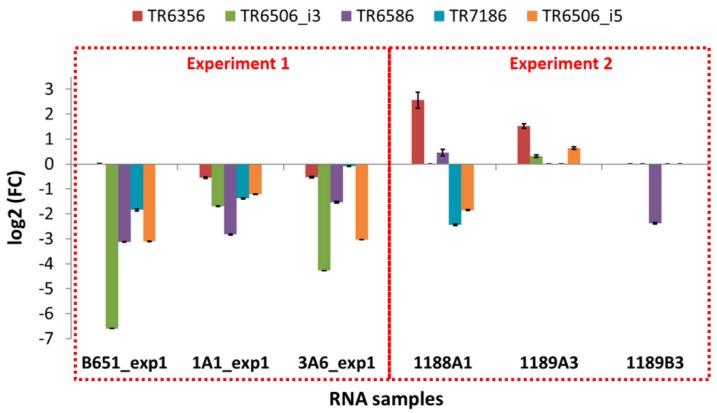
Gene expression results for the TEs for each sample of the two qPCR experiments; experiment 1 where the same strains used in the RNA-Seq and validation were used but acclimation time was altered, and experiment 2 where new *L. aporus* strains (1188A1, 1189A3, 1189B3) were isolated and used for qPCR after a short/very short acclimation period. The fold change is low temperature to high temperature expression values. Standard deviation values are shown.

**Figure 8:**
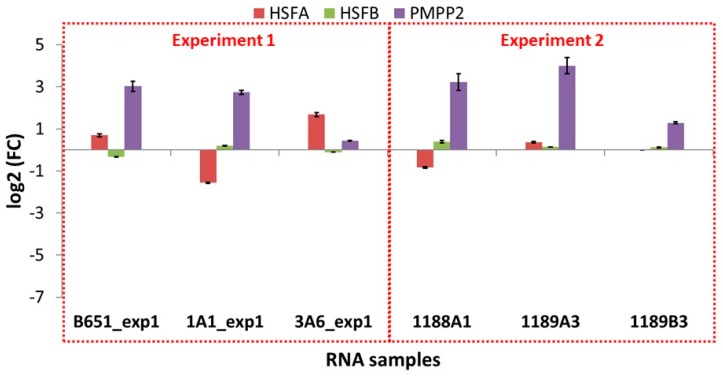
Gene expression results for the heat stress-related transcripts for each sample of the two qPCR experiments; experiment 1 where the same strains used in the RNA-Seq and validation were used but acclimation time was altered, and experiment 2 where new *L. aporus* strains (1188A1, 1189A3, 1189B3) were isolated and used for qPCR after a short/very short acclimation period. The fold change is low temperature to high temperature expression values. Standard deviation values are shown.

The expression of the selected transcripts was examined in independent experiments in which the exposure to high and low temperature was applied for a shorter time (approx. 41 vs. 155 days as an average), using the same strains as well as newly isolated ones. The results of these qPCR experiments (Figure 7 and Figure 8 and Table 10) were different from those of the RNA-Seq (Figure S2).

In B651, 1A1 and 3A6, the selected transcripts were downregulated at low temperature, while in the freshly isolated strain 1189B3—acclimated for the shortest time—all genes were either expressed at low levels or not detected. The only transcript that seemed to be unaffected from the acclimation duration and the time since isolation was SYM1, which was found to always be upregulated at low temperature.

On the other hand, looking at the freshly isolated strains 1188A1 and 1189A3 that were acclimated slightly more than 1189B3, most genes were again low or not expressed (TR6506_i3, TR6586, TR7186, HSFA, HSFB) but there were also upregulated TEs (TR6356 in both 1188A1 and 1189A3, TR6586 in 1188A1, TR6506_i3 and TR6506_i5 in 1189A3).

Amplification of the gene fragments related to TEs from the genomic DNA of the strains used in these experiments revealed that some of the elements were absent from the genome of strain 1189B3 (Figure S8), indicating that the absence of expression in this strain was due to the absence of the specific transposon in the genome. Similarly, in strain 1189A3, TR7186 appeared to be absent. In all other cases, amplification was successful.

### 3.8. Phylogenetic Analysis

RepeatMasker analysis on the five selected TE related transcripts (Table 10) revealed that two of them matched annotated repeats that were similar to CoDi/GyDi sequences. TR6506_i3 and GyDi2.1 both matched Gypsy3-I_TP, though at different regions each. TR7186 and the diatom-specific retrotransposon Blackbeard matched at the same region, Copia8-I_TP, but also at different ones (Table S3).

**Figure 9:**
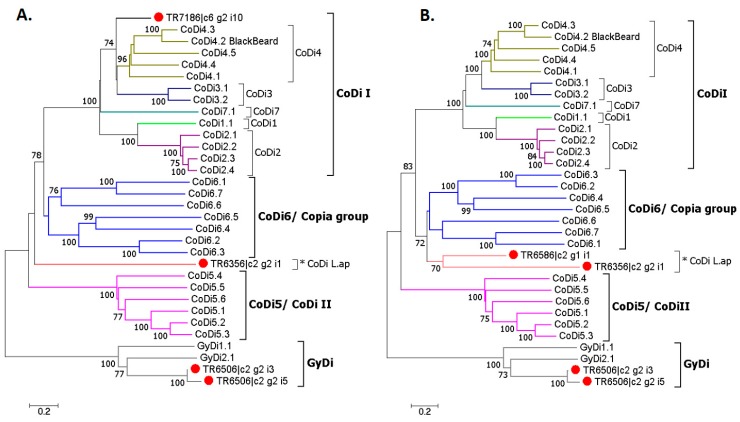
Neighbor-joining phylogenetic trees showing the relationships between the CoDis/GyDis and the selected transposable elements from *L. aporus* (GyDi group as outgroup). Bootstrap scores over 70% are shown. TR7186 (**A**) and TR6586 (**B**) were analysed separately since it was not possible to estimate their pairwise distance.

The phylogenetic tree gave a clearer picture about the relationship between the selected TEs and CoDis/GyDis. However, it was not possible to estimate the pairwise distance between TR7186 and TR6586 due to the much shorter protein sequence of the first, so two separate NJ trees were built (Figure 9). TR6506_i3 and TR6506_i5 clustered with the GyDi group, and TR7186 with the CoDi I group, while TR6586 and TR6356, named CoDi L.ap, were closely related to the Copia group.

## 4. Discussion

The analysis of *L. aporus* expression profiles at different temperatures based on several strains provided new information on the response of this species to cold stress, but also on the related intraspecific variability and its possible causes. In particular, we identified (i) specific genes, with a prominent role of TEs among them, that react to temperature changes and might be involved in acclimation and/or adaptation to environmental conditions, possibly underlying distinct phenological patterns, and (ii) high intraspecific variations in the species heat/cold response. The high numbers of DE genes only between the minimum and maximum temperature tested indicates that *L. aporus* mainly reacted to the low temperature, while it retained the same functional state at 26 °C and 19 °C. However, considering that one of the strains (B651) showed a very different reaction, this result could also be influenced by this high variance. A higher number of samples and replicates would be needed in order to completely clarify this point. In any case, the variability was much higher among strains, with 3622 DE genes, than among different experimental conditions, where only 276 DE genes were found, which highlights considerable intraspecific functional differences.

The functions related to the significant DE genes in response to temperature confirmed the stress reaction of *L. aporus* at low temperature. Oxidative, cold/heat, environmental or physiological stress responses, which seem to be coordinated by specific factors such as stress-activated kinases, AOX4, ALDHs, HSPs and HSFs, SYM1, MAI, FDH and transposable elements (TEs), were notable components of the species functional profile at 13 °C.

TEs proved to be the most significantly enriched units of the stress response, providing the first evidence for the possible role of TEs in the temperature stress response in *L. aporus*, as a few others have done recently in diatoms [47,82]. All TEs were of the LTR retrotransposon superfamily, confirming the tendency of relatively high abundance of LTR retrotransposons in diatom genomes [32]. Furthermore, the phylogenetic analysis showed that two of them belong to the GyDi group and one to the CoDi I group, which are both diatom-specific groups, while the CoDi L.ap group is related to the Copia group, which is also found in animals, plants and yeast.

When genes were grouped according to their expression profile in the different conditions, four out of the five TE-related transcripts that were selected for further analysis clustered with genes related to antioxidant activity. Antioxidant responses in plants are initiated after the accumulation of reactive oxygen species (ROS), such as hydrogen peroxide (H_2_O_2_) and hydroxyl free radical (·OH) [83,84]. The generation of numerous ROS can be linked to low temperature, when photosynthetic enzymes may be degraded and photo-damage may occur, leading to reduced photosynthetic activity and hence, the accumulation of excess energy [85]. Similarly, the overexpression of MAI at low temperature might mean that the mobilization of this gene in *L. aporus* contributes to genetic plasticity and finally adaptation to physiological stress in the same way that TEs do. Indeed, in bacteria, MAI undergoes frequent rearrangements, or else transposition events, under physiological stress conditions including prolonged storage at 4 °C or exposure to oxidative stress [81].

From the between-strains transcriptomic analysis, TEs again held a central role, with their expression showing strain-specificity as shown for fungi and plants [86,87,88]. The absence of specific TEs from the genomes of a couple of the strains (1189B3 and 1189A3), as well as the markedly different response to low, and for some genes, high, temperature of one of the three strains, B651, point to a high intraspecific variability. TE copy number variations (CNV) could also contribute to differences in expression patterns among strains, an issue that would be worth further investigation as CNV, if present, could be related to the strain response to cold. The clusters representation, among other things, allows one to appreciate the noise caused by B651. It is tempting to consider these differences as a result of adaptation: having been isolated from a summer population, strain B651 could be expected to perform worse at low temperature, with a lower expression level of genes involved in cold/heat response than the other two strains 1A1 and 3A6, belonging to winter populations. The clustering of B651 separately from 1A1 and 3A6 based on pathways including amino acid metabolism, glycolysis and gluconeogenesis, energy metabolism, cell growth and death and protein processing in endoplasmic reticulum could imply temperature adaptation changes in the baseline expression of key genes and pathways to maintain metabolic homeostasis, as highlighted in another centric diatom, *Chaetoceros* spp. [17]. The strains 1A1 and 3A6 could hence belong to a cold adapted population, though different from the one detected in the worldwide HTS study of the species [51].

Yet another hypothesis is that strain B651 was cultivated for four years longer than the other two strains, during which constant temperature conditions could have caused a shift of baseline gene expression and the downregulation or the loss of specific cold/heat response proteins. Indeed, the SNP analysis pointed at another peculiarity of the old strain, with an important loss of heterozygosity compared to the more recent ones, but also to the same strain back in 2011. This is not the first time that loss of heterozygosity is observed in diatom strains kept in prolonged cultivation [89]. The relevance of cultivation time would be supported by results of growth rate experiments performed on the other two strains isolated in the same year as B651 [51], which also showed worse performance under extreme temperatures. The effect of in-culture evolution could have been a more likely reason for their distinct behavior than the season of their occurrence, because these two strains were isolated in October and November and not in summer like B651, and were kept in the same conditions as B651 for long time. Stable temperature, light conditions and culture medium, along with the absence of any kind of competition or threat, for four years (more than 1000 generations) could have led to the divergence of B651 from its original ‘natural’ state and affected its ability to respond to temperature changes and stress. The loss of certain stress response mechanisms resulting in the very low expression of TEs and stress-related genes could be one of those effects. A strain of a pennate diatom of the genus *Pseudo-nitzschia* also displayed contrasting gene expression patterns under different nutrient conditions compared to earlier experiments [90], leading to the idea of in-culture progressive physiological modifications targeting functions that are energetically costly and confer no advantage in culture. In the *L. aporus* case, the SNP analysis points to a genetic modification following the physiological one, but we prefer to remain cautious about this assumption since genotypes defined only on RNA data can be a result of several confounding pre- and post-transcriptional alternative roads (monoallelic expression, allelic imbalance, DNA editing, RNA editing, etc.). Finally, in support of the in-culture evolution hypothesis, it should also be noted that after two years from isolation, the two strains 1A1 and 3A6 did not show any upregulation, whereas the freshly isolated strains 1188A1 and 1189A3 did show upregulation in a couple of their TEs, even after a short acclimation at low temperature. This small yet existent difference indicates that the absence of stress response is not strain-related to B651 alone, but, when compared with more freshly isolated strains, the same observation can be made for all strains maintained in culture.

In addition to intraspecific diversity and possible in-culture evolution, the response to a stressful environment can also vary in relation with the duration of the exposure to stressful conditions. The contrasting expression patterns noticed in the most recently cultured strains that underwent a shorter period of acclimation indicate a possible role of the exposure time to the stressing factor in *L. aporus*: transcripts related to cold stress were not at all expressed (with 22 days acclimation), or, with a few exceptions, expressed at a very low level (with 26–43 days acclimation). Similar changes were seen in the cold water coralline algae *Lithothamnion glaciale*, where two phases were identified in a long term experiment under the stress of elevated CO_2_ treatment: the “passive” phase during the first 3 months and the “active” phase by the end of 10 months when energy was allocated from cell growth to structural support, showing a clear adaptive plasticity response of the algae [91]. It therefore seems that the “passive” phase in *L. aporus* lasts about 10–20 days, after which these TEs are expressed. Nevertheless, the response to a short and long-term changing environment may be not only species- but also strain-specific [92], and the relative importance of strain-specific reaction versus time of exposure to stress is hard to disentangle in this study as the strains isolated more recently were also the ones that underwent the shorter acclimation.

## 5. Conclusions

Variability within species is a relevant issue especially when studying the potential for adaptation of phytoplankton, which instead is often extrapolated from single strain responses of representative species. In the present study, gene expression patterns of the warm water diatom *L. aporus* have revealed profound differences among strain responses, which were as important, if not of greater importance, than those among temperature conditions. In addition, time spent by the strains under stable conditions and in-culture evolution seem to play an important role in the differences observed.

TE-related sequences appear to play a role in the adaptation to cold conditions, but they may get silenced when the cells remain for a long period in the same environmental conditions. This result points at the possible role of TEs in the generation of the phenotypic plasticity that can lead to genetic diversity and ultimately to the success of diatoms under different and variable environmental conditions. More experiments are needed to demonstrate the actual importance of TEs in the response of *L. aporus* to stress, e.g., through the assessment of methylation, which is thought to be involved in heterochromatin formation and maintenance that controls TE mobility. The role of the TEs of interest in transcriptional regulation under cold stress should also be investigated, while experimental evolution approaches or single cell based strategies targeting specific TEs could provide evidence of de novo TE insertions over different time scales and of any changes in expression that can improve fitness.

## Figures and Tables

**Table 1:** *Leptocylindrus aporus* strains isolated from the Gulf of Naples and used for RNA extraction, sequencing and qPCR experiments.

#	Strain Code	Isolation Date	Temperature	RNA-Seq	qPCR
1	B651	21 August 2010	13 °C	X	X
			19 °C	X	X
			26 °C	X	X
2	1A1	20 December 2013	13 °C	X	X
			19 °C	X	X
			26 °C	X	X
3	3A6	28 January 2014	13 °C	X	X
			19 °C	X	X
			26 °C	X	X
4	1188A1	4 February 2016	13 °C		X
			19 °C		X
			26 °C		X
5	1189A3	19 February 2016	13 °C		X
			19 °C		X
			26 °C		X
6	1189B3	19 February 2016	13 °C		X
			19 °C		X
			26 °C		X

**Table 2:** Start dates and duration of acclimation for each sample used in RNA-Seq.

Strain	Temperature (°C)	Acclimation Start Date (dd/mm/yy)	Acclimation Duration (Days/~Cell Cycles)
B651	13	01 September 2014	140/~70
	19		150/~150
	26		203/~203
1A1	13		205/~102
	19		105/~105
	26		105/~105
3A6	13		142/~71
	19		105/~105
	26		135/~135

**Table 3:** Selected TE- and heat- related transcripts and reference genes for qPCR, their corresponding encoding domains and primer sequences.

Transcript ID	Gene/Domain Name to be Amplified	Primer Sequences (5′–3′)
TR6356|c2_g2_i1	RNase HI RT Ty1/Copia family	Fwd: GTAGCACGGAGGCGGAATTA
		Rev: GCTCTTTCTCCCGTTCGTCT
TR7186|c6_g2_i10	RNase HI RT Ty1/Copia family	Fwd: CGCGAGTCATGCCACTAATC
		Rev: AACCCCAGTCCTTAATGCCA
TR6586|c2_g1_i1	RVT 2 Reverse transcriptase	Fwd: CACTCGATGCAAGCAAGTCG
		Rev: CCCCTTTGATGAGTGCGTCT
TR6506|c2_g2_i3	rve Integrase core domain (Ty3/Gypsy family)	Fwd: TGGCCGAAGTACAGGACCTA
		Rev: ATTGGCCTGAGGGTTTCGAG
TR6506|c2_g2_i5	rve Integrase core domain (Ty3/Gypsy family)	Fwd: AGAGAGCGGACGAAATAGCG
		Rev: ACAATTACGTGCTGAGGCCA
TR264|c0_g1_i1	Heat Shock Factor A-1a (HSFA1a)	Fwd: ACCATGGGGCAACCAAGATA
		Rev: GTGGGGAGATTTCGGCCATT
TR936|c0_g1_i1	Heat Shock Factor B-2a (HSFB2a)	Fwd: GTCGTCGTTTCGTAAGCAGC
		Rev: CAGCTTGGGCATTCCTCGTA
TR1252|c0_g1_i1	Stress-induced Yeast ortholog of Mpv17 (SYM1)	Fwd: TGTTGGGGTATATGGATACCAGT
		Rev: TTCGGAGAAACTCTGGAACAA
Reference gene 1	TUBA1	Fwd: TCAATTCGGGACAGTGCCTC
		Rev: GCCAATGTTCCTGGTGGAGA
Reference gene 2	TUBB6	Fwd: GGTAGAGAACGCGGACCAAT
		Rev: TTGTCCGGGGAACCTCAAAG

**Table 4:** Start date and duration of acclimation for each sample used in the qPCR experiments.

Strain	Temperature (°C)	Experiment Number	Acclimation Start Date (dd/mm/yy)	Acclimation Duration (Days/~Cell Cycles)
B651	13	1	15 January 2016	60/~30
	19			40/~40
	26			47/~47
1A1	13			66/~33
	19			39/~39
	26			44/~44
3A6	13			65/~32
	19			46/~46
	26			50/~50
1188A1	13	2	4 March 2016	43/~22
	19			17/~17
	26			34/~34
1189A3	13		11 April 2016	26/~13
	19			8/~8
	26			17/~17
1189B3	13		15 April 2016	22/~11
	19			9/~9
	26			19/~19

**Table 5:** Number of significantly DE genes between low (13 °C) and high (26 °C) and low and medium (19 °C) temperature in *L. aporus*. No significant DE genes were found between medium and high temperature.

	Significant DEs at Low vs. High Temperature	Significant DEs at Low vs. Medium Temperature
**Upregulated**	243	8
**Down regulated**	33	1
**Total**	276	9

**Table 6:** Annotation of significantly DE transcripts related to temperature and stress. The fold change (FC) is low to high temperature expression values. FC values are more than one for proteins that were represented by more than one transcript.

Proteins	HSPs *	Log2(FC)	Related Functions
Peroxiredoxins (PRXs)	Peroxiredoxin Q	3.5	Antioxidant-oxidoreductase activity, response to oxidative stress
	Peroxiredoxin-6	4.32	
	1-Cys peroxiredoxin A	4.94	
Ferredoxin-NADP reductase		3.35	
Aldehyde Dehydrogenases (ALDHs)	Probable aldehyde dehydrogenase	3.36	
	Methylmalonate-semialdehyde dehydrogenase	3.54	
Ubiquinol Oxidase 4 Chloroplastic/Chromoplastic (AOX4)		4.66	
Serine/threonine-protein kinases	SAPK2	−4.61	Stress activated kinases. Regulation of cellular processes like proliferation, division, survival, metabolism & cell-cycle progression
	fhkD	3	
Chaperones	E3 ubiquitin-protein ligase RNF181	−3.68	Heat stress response
	Peptidyl-prolyl cis-trans isomerase FKBP2	3.01	
	Putative lysine-specific demethylase JMJD5	2.7	
	ATPase family AAA domain-containing protein 3-B	3.9	
Heat Shock Proteins and factors (HSPs and HSFs)	Heat shock factor protein 1	−4.37	
	Heat stress transcription factor C-1b	−3.34	
	Heat stress transcription factor B-2a	3.31	
	Heat stress transcription factor A-1a	5.16	
Photosystem II 12 kDa extrinsic protein (PSBU)		4.41	
Stress-induced Yeast ortholog of the mammalian Mpv17 (SYM1)		3.95	
Ribosome-associated inhibitor A (RaiA)		3.26	Response to environmental or physiological stress such as cold shock
Formate dehydrogenase (FDH)		3.96	
Proteins belonging to the magnetosome island (MAI)	mgI388	4.11	
	mgI382	4.91	
Ribonuclease and/or Reverse transcriptase		5.42, 5.61, 3.75, 2.72, 3.12, 3.23, 3.56, 3.99, 4.11	DNA integration
gag-pol polyprotein		6.66	
Integrase		3.46, 4.11	

* High-scoring segment pairs.

**Table 7:** Statistics calculated based on SNPs called on strains 1A1, 3A6 and B651 at 19 °C using the transcriptome assembled in this study. Total SNPs refer to single nucleotide polymorphisms and indels (insertions and deletions).

	1A1	3A6	B651
**SNPs**	35,195	36,756	69,302
**SNP Transitions/Transversions**	1.74 (33,491/19,193)	1.75 (35,923/20,536)	1.79 (79,089/44,307)
**Total Het/Hom ratio**	1.03 (20,835/20,325)	0.88 (20,072/22,719)	0.30 (18,291/61,028)
**SNP Het/Hom ratio**	1.02 (17,745/17,450)	0.87 (17,107/19,649)	0.28 (15,268/54,034)

**Table 8:** Statistics calculated based on SNPs called on B651 sampled in 2011 (MMETSP) and in 2014, at 19 °C using the transcriptome available in MMETSP. Total SNPs refer to single nucleotide polymorphisms and indels (insertions and deletions).

	B651 MMETSP (2011)	B651 (2014)
**SNPs**	29,260	24,967
**SNP Transitions/Transversions**	1.74 (19,915/11,421)	1.74 (17,938/10,289)
**Total Het/Hom ratio**	11.27 (32,400/2,874)	6.12 (25,735/4,208)
**SNP Het/Hom ratio**	13.22 (27,203/2,057)	6.70 (21,726/3,241)

**Table 9:** Selected TE-related transcripts, their corresponding RepeatMasker annotation and RNA-Seq expression values (CPM values) for each sample.

ID	Repeat Masker Annotation	B651			1A1			3A6		
		13 °C	19 °C	26 °C	13 °C	19 °C	26 °C	13 °C	19 °C	26 °C
TR6356	Copia-1-I FCy *	36.23	8.73	5.69	333.9	71.38	41.21	468.23	100.02	83.15
TR7186	Copia8-I TP *	0	0	0	16.96	2.81	1.08	35.43	9.39	2.81
TR6586	-	31.47	18.53	3.17	0	0	0	0.47	0.06	0
TR6506_i3	Gypsy3-I TP	0.25	0.06	0	24.3	1.57	0.05	52.46	7.68	3.63
TR6506_i5	-	0.38	0.06	0	17.61	0.98	0.098	43.52	6.77	3.05

* FCy: *Fragilariopsis cylindrus*, TP: *Thalassiosira pseudonana*.

**Table 10:** Expression of selected transcripts in samples used for qPCR. Strains B651, 1A1 and 3A6 were acclimated for approx. 55 days on average, while strains 1188A1, 1189A3 and1189B3 were acclimated for approx. 27 days on average. Green shading is for presence, orange shading for very low expression and red shading for absence.

Strain	B651			1A1			3A6			1188A1			1189A3			1189B3		
T (°C)	13	19	26	13	19	26	13	19	26	13	19	26	13	19	26	13	19	26
**TR6356**	+	+	+	+	+	+	+	+	+	+	+	+	+	+	+	-	-	-
* TR6506_i3	!	+	+	+	+	+	+	+	+	(!)	(!)	(+)	(!)	(!)	(!)	-	-	(!)
**TR6506_i5**	+	+	+	+	+	+	+	+	+	(+)	(+)	(!)	+	+	+	-	(!)	(!)
* TR6586	+	+	+	!	!	!	+	!	!	(!)	(!)	(+)	-	-	-	-	-	(!)
* TR7186	!	!	!	+	+	+	+	+	+	(!)	(!)	(+)	-	-	-	-	-	-
**HSFA**	(+)	(+)	(+)	+	+	+	+	+	+	(+)	(+)	(+)	+	-	+	-	-	-
**HSFB**	+	+	+	(+)	(+)	(+)	+	+	+	+	+	+	(+)	(+)	(+)	(+)	(+)	(+)
**SYM1**	+	+	+	+	+	+	+	+	+	+	+	+	+	+	+	+	+	+

(+): presence, +: presence, in agreement with RNA-Seq; (!): very low expression (ct>=34< ctnegative), !: very low expression (ct>=34< ctnegative), in agreement with RNA-Seq; -: absence (samples with ct>ctnegative were as well considered absent); *: Significant differential expression for B651 (against 1A1 or 3A6) validated.

## Supplementary Materials

The following are available online at https://www.mdpi.com/2073-4425/11/1/9/s1, Figure S1: Acclimation scheme of *L. aporus* strains at the three different temperatures, Figure S2: Agarose gel of the gradient PCR, Figure S3: qPCR amplification plot and melting curves, Figure S4: NMDS analysis, Figure S5: Heatmap of key genes and pathways, Figure S6: qPCR validation results for the TEs, Figure S7: qPCR validation results for the heat stress-related transcripts, Figure S8: Amplification of the TE-related gene fragments from genomic DNA, Table S1: Transcriptome statistics, Table S2: Comparison of present transcriptome with MMETSP transcriptome, Table S3: RepeatMasker results of TE related transcripts, Supplementary File 1: Growth experiment results, Supplementary File 2: Significantly DE transcripts between low and high T, Supplementary File 3: Significantly DE transcripts between low and medium T, Supplementary File 4: significantly DE transcripts between 1A1 and 3A6; Supplementary File 5: Significantly DE transcripts between 1A1 and B651, Supplementary File 6: Significantly DE transcripts between B651 and 3A6, Supplementary File 7: Explanation of *Annocript* output columns present in Suppl.Files 2-6.
