# Supplementary material for: Intraspecific Diversity in the Cold Stress Response of Transposable Elements in the Diatom Leptocylindrus aporus
Source: Genes (Basel). 2019 Dec 20;11(1):9. doi: 10.3390/genes11010009 (PMC7017206; doi:10.3390/genes11010009)
Supplement: Supplementary file 1 [file genes-11-00009-s001.zip › Supplementary File 1.docx]

**Supplementary Results**

The available *L. aporus* strains were the following:

1. B651, isolated on 21/8/2010.
2. B704, isolated in October of 2009.
3. B764, isolated on 18/11/2010.
4. 1A1, isolated on 20/12/2013.
5. 3A6, isolated on 28/01/2014
6. 1089-10, isolated on 14/01/2014.

Two of the *L. aporus* strains, B651 and B764, which were maintained in the SZN collection for long time (since 2010) seemed to need more time to acclimatize to the temperature of 26 °C compared to the rest of the strains and needed one more growth cycle. Finally, the growth rate for all strains remained consistent for at least three sequential growth curves (Table 1). Despite the fact that some growth rates were still significantly different in the last two growth cycles, these strains were considered acclimatized because of a much smaller deviation compared to previous measurements (Table 1).

Table 1. Average between duplicates of growth rates, k (div./day), for *L. aporus* strains at the three different growth temperatures inferred from fluorescence values during acclimatization. The strains with the significantly different (Welch and Brown-Forsythe ANOVA, p<0.05) growth rates in the last two growth rates are written in red.

| **Strain** | **1^st^ Growth Curve** | **2^nd^ Growth Curve** | **3^rd^ Growth Curve** |
| --- | --- | --- | --- |
| **B651 (13^o^C)** | 0.42 | 0.45 | 0.40 |
| **B651 (19 °C)** | 0.83 | 0.92 | 0.86 |
| **B651 (26 °C)** | 0.94 | 1.06 | 0.85 |
| **B704 (13 °C)** | 0.38 | 0.38 | 0.45 |
| **B704 (19 °C)** | 0.94 | 1.07 | 0.98 |
| **B704 (26 °C)** | 0.84 | 1.15 | 1.45 |
| **B764 (13 °C)** | 0.36 | 0.37 | 0.52 |
| **B764 (19 °C)** | 0.91 | 1.12 | 1.06 |
| **B764 (26 °C)** | 0.83 | 1.04 | 0.91 |
| **1A1 (13 °C)** | 0.42 | 0.50 | 0.50 |
| **1A1 (19 °C)** | 1.05 | 0.94 | 0.90 |
| **1A1 (26 °C)** | 0.98 | 1.11 | 0.97 |
| **1089-10 (13 °C)** | 0.49 | 0.51 | 0.53 |
| **1089-10 (19 °C)** | 1.04 | 1.23 | 1.14 |
| **1089-10 (26 °C)** | 1.11 | 1.28 | 1.23 |
| **3A6 (13 °C)** | 0.50 | 0.46 | 0.50 |
| **3A6 (19 °C)** | 1.21 | 1.10 | 0.98 |
| **3A6 (26 °C)** | 1.00 | 1.02 | 0.96 |

The growth curves during the growth experiment for all strains showed a much longer growth cycle at 13 °C compared to the other temperature conditions, while at the same time there was a high intraspecific variability in growth and duration of the cycle under all temperatures conditions (Fig. 1).


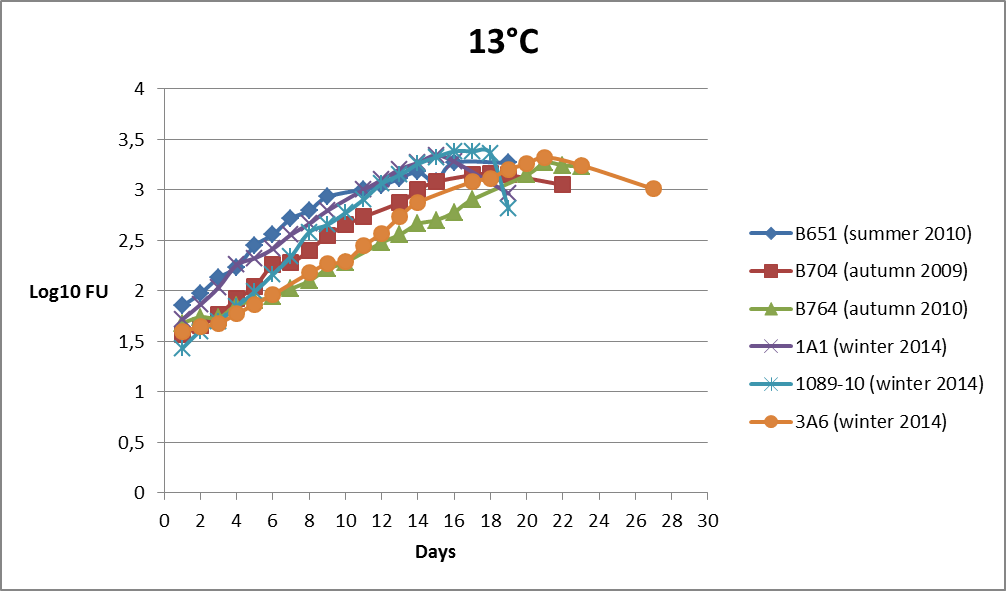

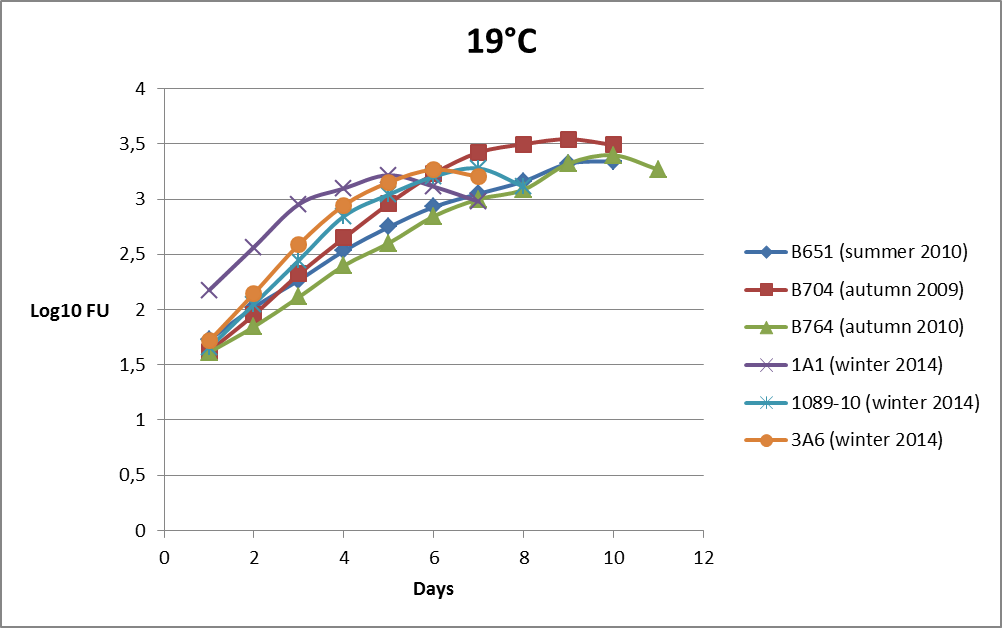


**B.**

**A.**


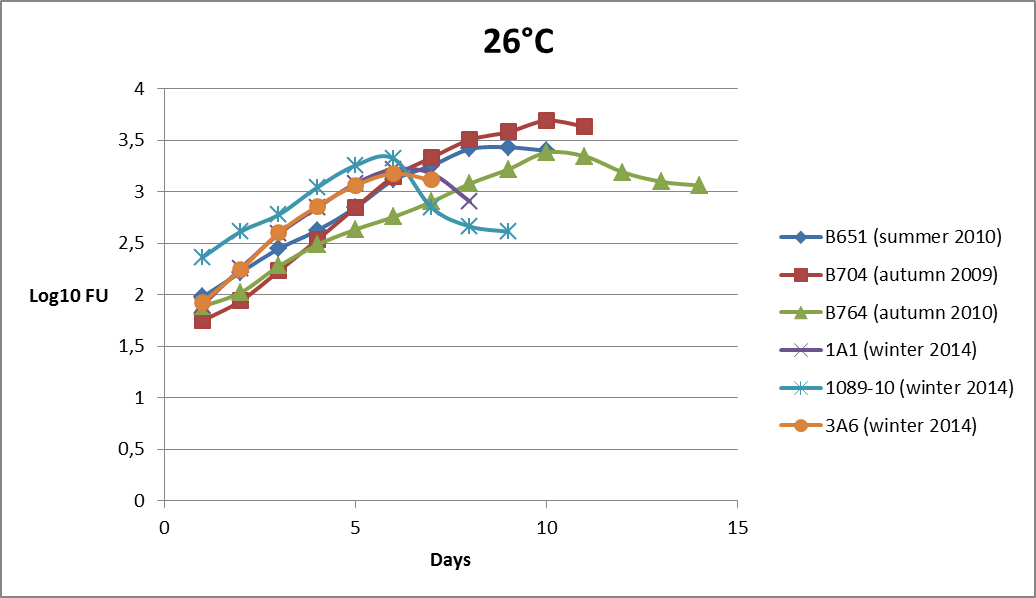


**C.**

Figure 1. *Leptocylindrus aporus* strains growth curves established by daily arbitrary fluorescence measurements at three different temperatures, 13 °C (A), 19 °C (B), 26 °C (C). In the legend, the season of isolation of each strain is indicated in brackets.

There were several cases where the growth rate during the experiment deviated considerably from the one observed during the acclimatization period, as shown by the high standard deviations of the growth averaged for B651 at 19 °C and 26 °C, B704 at 26 °C, B764 at 19 °C and 26 °C, 1089-10 at 26 °C and 3A6 at 13 °C (Fig. 2). The growth rate was significantly different between low temperature and medium/ high temperature (Welch ANOVA, Games Howell post hoc test; p<0.05) within and among all strains.


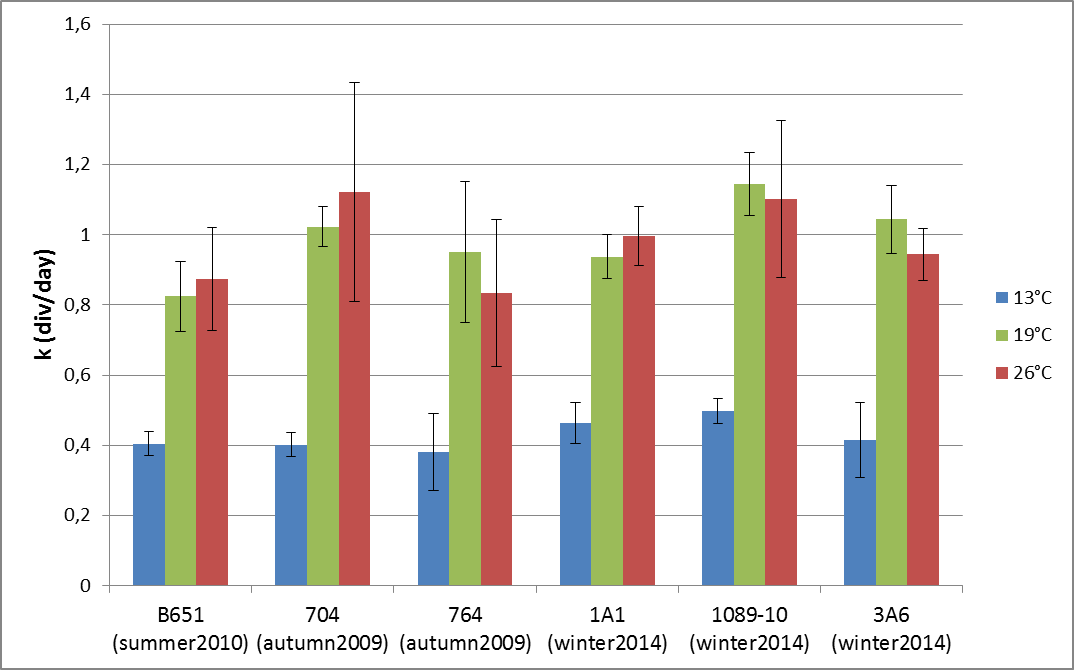


Figure 2. Bar graph of growth rates of *L. aporus* strains at three different growth temperatures, based on counts. Standard deviation values were calculated based on growth rate values of the four last growth curves (the last three cycles of acclimatization plus the growth experiment cycle). In the legend to the x axis, the season of isolation of each strain is indicated in brackets.

Regarding the cell size and morphology, all strains appeared to form longer colonies and the percentage of larger cells increased at medium and high temperature, with the recently isolated strains forming longer chains (Table 2). No significant differences were detected between strains isolated in different seasons (ANOVA tests, p>0.05).

Table 2. Size (diameter and pervalvar distance) for 25 randomly selected cells and colony (chain length) condition for *L. aporus* strains at the three different growth temperatures.

| **Strain** | **T (^o^C)** | **Mean Diameter (μm)** | **Pervalvar axis (μm)** | **% of cells with diameter >= 8 μm** | | **Max. diam. (μm)** | **Min. and Max. Chain Length** | |
| --- | --- | --- | --- | --- | --- | --- | --- | --- |
| **B651 (summer 2010)** | **13** | 5 | 15 | | 0 | 5 | | Single and up to 5 cells |
|  | **19** | 7 | 15 | | 0 | 7 | | Single and up to 10 cells |
|  | **26** | 5 | 15 | | 37,5 | 10 | | Single and up to 12 cells |
|  |  |  |  | |  |  | |  |
| **B704 (autumn 2009)** | **13** | 8 | 15 | | 100 | 8 | | Single and up to 6 cells |
|  | **19** | 5 | 10 | | 0 | 5 | | Single and up to 7 cells |
|  | **26** | 5 | 10 | | 0 | 5 | | Single and up to 11 cells |
|  |  |  |  | |  |  | |  |
| **B764 (autumn 2010)** | **13** | 7 | 15 | | 0 | 7 | | Single and up to 6 cells |
|  | **19** | 5 | 15 | | 20 | 8 | | Single and up to 11 cells |
|  | **26** | 5 | 15 | | 10 | 8 | | Single and up to 16 cells |
|  |  |  |  | |  |  | |  |
| **1A1 (winter 2014)** | **13** | 7,5 | 15 | | 20 | 10 | | Single and up to 22 cells |
|  | **19** | 7,5 | 15 | | 50 | 10 | | Single and up to 25 cells |
|  | **26** | 7,5 | 15 | | 45 | 10 | | Couplets and up to 24 cells |
|  |  |  |  | |  |  | |  |
| **1089-10 (winter 2014)** | **13** | 5 | 15 | | 0 | 5 | | Couplets and up to 8 cells |
|  | **19** | 5 | 15 | | 35 | 20 | | Single and up to 20cells |
|  | **26** | 5 | 10 | | 0 | 5 | | Couplets and up to 11 cells |
|  |  |  |  | |  |  | |  |
| **3A6 (winter 2014)** | **13** | 4 | 13 | | 0 | 4 | | Single and up to 11 cells |
|  | **19** | 5 | 15 | | 17 | 10 | | Single and up to 11 cells |
|  | **26** | 10 | 13 | | 5 | 20 | | Triplets and up to 54 cells |
